# Medical Items

**Published:** 1898-03

**Authors:** 


### *MEDICAL ITEMS.*

---

Dr. Robert Jones, of Rosalie, Texas, is coming East for a trip this spring.

---

The Journal had the pleasure of a visit recently from Dr. Jas. T. Lovvorn, of Bowden, Ga.

---

Dr. Montague L. Boyd, of Savannah, died February 15th, from septic infection acquired in an operation.

---

Dr. H. T. Whitney, Medical Missionary in China, is engaged in translating Gray's Anatomy into the Chinese language.

---

Dr. E. C. Seguin, of New York, the well-known author and authority on mental and nervous diseases, died February 19th, in his fifty-fifth year. The immediate cause of his death was carcinoma of the liver.

---

It is said that one person out of eight hundred is blind to the *X*-rays; that is, when looking through the fluoroscope they cannot see the bones of the hand, coins in a book, etc. This is no doubt analogous to color-blindness.

---

A Frenchman has discovered that the "poison of the hornet renders one immune to that of the viper." People, however, will be slow to accept the conclusion that the hornet sting offers any particular advantages over other more agreeable and time-honored remedies for snake-bite.

DR. THEOPHILUS PARVIN, of Philadelphia, died January 29th, from pulmonary edema due to renal disease. Dr. Parvin was sixty-eight years of age, and for many years had been Professor of Obstetrics in the Jefferson Medical College. He was author of a popular text-book on obstetrics.

---

IN a late *Philadelphia Medical Journal* Dr. Osler, of Baltimore, pays his respects to the Woodbridge treatment of typhoid fever. He is greatly impressed, he says, in reading Dr. Woodbridge's articles upon typhoid, with the "crude, unscientific character of his work, and with the ignorance everywhere displayed of the nature of typhoid fever, and with the specific vaunting of a cure-all for it."

---

WHAT is a "Specialist in Practical Medicine?" That is the way a writer signs himself in a more or less esteemed contemporary. This is a little more elegant and not so common as the well-worn "Specialist in all medical and surgical diseases of men, women, and children," besides enabling the proprietor of the term to embrace in his comprehensive specialty hygiene and sanitary science, life insurance examination, railway surgery, drug habits, toxicology, and everything else, from lost manhood to leprosy. What further indignities await the once honorable name of specialist remain to be seen.

---

DR. F. E. DANIEL, editor of the Texas *Medical Journal* and late Surgeon C. S. A., has written a book, "The Recollections of a Rebel Surgeon," or "In the Doctor's Sappy Days." It will soon be published, and will be a "series of short sketches, personal reminiscences, mostly humorous, of life in camp, field and hospital, "endurin' of the war." Dr. Daniel says the work will be "real good," and, from our acquaintance with other works of the doctor's bright and incisive pen, we are willing to accept this short criticism in advance. We venture to predict that there will not be a dull page in the book. Look out for it, doctor; then get it and read it.

---

CHARLES TODD QUINTARD, M.D., D.D., LL.D., Bishop of the Protestant Episcopal Diocese of Tennessee, died February 15th, at Darien, Ga., of heart-failure. He was born in Stamford in 1824. After leaving Trinity School he studied medicine with Dr. James R. Wood and Dr. Valentine Mott, and was graduated from the University of the City of New York in 1847. He then removed to Athens, Ga., where he began the practice of medicine. In 1851 he was appointed professor of physiology and pathologic anatomy in the medical college at Memphis, Tenn. In 1875 he was appointed a deacon in the Protestant Episcopal Church, and in January, 1857, became rector of the Calvary Church, Memphis. In 1865 he was elected Bishop of Tennessee.—*Ex.*

---

WE imagine that the editor of the *Pacific Medical Journal* is off on his vacation, having left the office boy in charge, armed with a pair of shears and a breezy western newspaper. The last number of our usually esteemed contemporary conveys the following bits of wonderful misinformation:

"A Missouri girl recently had a cataract burned from her eye by the popping of some hot grease in her face and eye. She bandaged the burn for a few days with tea leaves, and on removing the bandage, found she could see as well as ever."

"A young man of Rahway, Pa., whose weight had decreased from 145 to 90 pounds, and who was being treated for consumption, coughed up a live frog three inches long, which is in the possession of the physician, who suspects the presence of other batrachains somewhere about his patient."

A "NATIONAL Society for the Study of Epilepsy and the Care and Treatment of Epilepsy" is proposed by Dr. W. P. Spratling, Medical Superintendent of the Craig Colony of Epileptics, Sonyea, N. Y. The objects of such a society would be: 1. The scientific study of epilepsy. 2. The rational treatment of the disease. 3. The best methods of caring for dependent epileptics, including, (*a*) The construction of proper homes, based upon a study of the epileptic's needs as to classification and environment. (*b*) The study of the utilization of the epileptic's labor, for economic, scientific and ethical reasons. (*c*) The study of the best educational methods to be employed, including manual, industrial, intellectual and moral forms and forces.

It is thought that an effort will be made to organize the society in May next.

---

AN army medical examining board will be in session at Washington, D. C., during the month of May for the examination of candidates for appointment to the Medical Corps of the United States Army, to fill existing vacancies. Persons desiring to present themselves for examination will make application to the Secretary of War before April 15, 1898, for the necessary invitation, giving the date and place of birth, the place and State of permanent residence, the fact of American citizenship, the name of the medical college from which they were graduated, and a record of the service in hospital, if any, from the authorities thereof. The application should be accompanied by certificates based on personal acquaintance, from at least two reputable persons, as to his citizenship, character and habits. The candidate must be between 22 and 29 years of age, and a graduate from a Regular Medical College, as evidence of which his diploma must be submitted to the Board. Further information regarding the examinations may be obtained by addressing the Surgeon-General U. S. Army, Geo. M. Sternberg, Washington, D. C.

